# Hearing Care Access for those with Dementia in Primary Care Practice: Identifying Opportunities to Improve Integration

**DOI:** 10.1002/alz70858_099370

**Published:** 2025-12-25

**Authors:** Danielle Powell, Lindsey Hoffman, Afia Obeng, Ariella Sapoznick, Martha Abshire Saylor, Stephanie Nothelle, Esther S. Oh, Jennifer L Wolff, Nicholas S Reed

**Affiliations:** ^1^ University of Maryland, College Park, MD, USA; ^2^ Johns Hopkins University, Baltimore, MD, USA; ^3^ Johns Hopkins School of Medicine, Baltimore, MD, USA; ^4^ Johns Hopkins University School of Medicine, Baltimore, MD, USA; ^5^ Johns Hopkins Bloomberg School of Public Health, Baltimore, MD, USA; ^6^ New York University, New York, NY, USA

## Abstract

Current care delivery models for hearing and hearing health have little focus on how to adequately equip primary care providers in initiating the hearing care process following identified concerns. Current recommendations on how to manage hearing loss for older adults with cognitive concerns provide minimal insight for primary care providers on how to incorporate hearing conversations amidst other care needs. We used thematic analysis to analyze transcripts of semi‐structured interviews with primary care providers (*n* = 6) regarding facilitators and barriers to hearing care for older adults with dementia. Results indicate providers universally recognized limitations in their ability to support hearing concerns— especially for those with complex care needs. We identified 5 primary themes: 1) primary care providers recognize the importance of hearing for those with dementia to support social engagement; 2) providers do not feel equipped to engage in hearing care conversations or distinguish between impaired hearing vs cognitive impairment in patients; 3) other health needs supersede hearing unless time and patient interest allows; and 4) hearing care process resources are desired to equip providers to meet initial indication of hearing concerns; 5) information on alternative options for hearing care in those with dementia is vital to provide effective care. Our findings reinforce the need to identify strategies and structured resources which support primary care provider's ability to attend to hearing concerns within existing workflows for those with dementia to enable higher quality and more connected care.

